# Supplementary material for: Longitudinal trajectories, correlations and mortality associations of nine biological ages across 20-years follow-up
Source: eLife. 2020 Feb 11;9:e51507. doi: 10.7554/eLife.51507 (PMC7012595; doi:10.7554/eLife.51507)
Supplement: Supplementary file 1. — (A) Number of individuals with information on BAs by IPTs. (B) CA-biomarker correlations in the development of physiological age. (C) List of the 42 items included in the FI and their scoring. (D) Characteristics of baseline (first available) complete measurements in 288 individuals. (E) Repeated measures correlation coefficients of BAs in 288 individuals. (F) Survival analyses of baseline (first available) BA residuals with the risk of all-cause mortality in 845 individuals (one-BA models). (G) Survival analyses of baseline BAs with the risk of all-cause mortality in 845 individuals stratified by sex (one-BA models). (H) Survival analyses of baseline BAs with the risk of all-cause mortality in 845 individuals stratified by age group (one-BA models). (I) Survival analyses of baseline BAs with the risk of all-cause mortality in 845 individuals stratified by smoking status (one-BA models). (J) Survival analyses of baseline BAs with the risk of all-cause mortality in 288 individuals with complete measurements (one-BA models). (K) Survival analysis with additional adjustment for previous diseases. [file elife-51507-supp1.docx]

**Supplementary file 1A. Number of individuals with information on BAs by IPTs**

|  | IPT | | | | | | | | |
| --- | --- | --- | --- | --- | --- | --- | --- | --- | --- |
|  | 1 | 2 | 3 | 5 | 6 | 7 | 8 | 9 | 10 |
| Years of investigation | 1986-1988 | 1989-1991 | 1992-1994 | 1999-2001 | 2002-2004 | 2005-2007 | 2008-2010 | 2010-2012 | 2012-2014 |
| Telomere length | 0 | 0 | 367 | 387 | 293 | 0 | 297 | 255 | 0 |
| DNAmAge (4 types) | 0 | 0 | 247 | 244 | 192 | 0 | 190 | 155 | 0 |
| Physiological age | 376 | 524 | 504 | 493 | 372 | 183 | 273 | 239 | 211 |
| Cognitive function | 592 | 480 | 463 | 450 | 362 | 271 | 217 | 210 | 0 |
| FAI | 0 | 541 | 453 | 476 | 374 | 333 | 286 | 237 | 222 |
| FI | 0 | 570 | 479 | 521 | 419 | 367 | 306 | 262 | 238 |
| At least one BA | 604 | 590 | 565 | 535 | 437 | 371 | 331 | 294 | 246 |
| Complete nine BA | 0 | 0 | 122 | 178 | 120 | 0 | 106 | 86 | 0 |

DNAmAges include 4 different types: Horvath, Hannum, PhenoAge, GrimAge.

BA, biological age; IPT, In-person testing; DNAmAge, DNA methylation age; FAI, functional aging index; FI, frailty index.

**Supplementary file 1B. CA-biomarker correlations in the development of physiological age**

| 19 candidate biomarkers which were assessed in all 9 IPTs | | Men | |  | Women | |
| --- | --- | --- | --- | --- | --- | --- |
|  |  | r | Included |  | r | Included |
| Physical examination | BMI (kg/m^2^) | -0.157 | ✓ |  | 0.064 |  |
|  | Waist-hip ratio (WHR) | -0.183 | ✓ |  | 0.098 |  |
|  | Weight (kg) | -0.266 | ✓ |  | -0.094 |  |
|  | Waist circumference (cm) | -0.044 |  |  | 0.156 | ✓ |
|  | Hip circumference (cm) | 0.056 |  |  | 0.121 | ✓ |
|  | Systolic blood pressure (mmHg) | 0.198 | ✓ |  | 0.300 | ✓ |
|  | Diastolic blood pressure (mmHg) | -0.117 | ✓ |  | -0.001 |  |
|  | Pulse (/min) | -0.073 |  |  | 0.007 |  |
| Urine test | Albumin (Urine strip test) | 0.033 |  |  | -0.039 |  |
|  | Erythrocyte (Urine strip test) | 0.055 |  |  | 0.027 |  |
|  | Ketone (Urine strip test) | -0.063 |  |  | 0.060 |  |
|  | Glucose (Urine strip test) | -0.036 |  |  | 0.059 |  |
| Blood test | Hemoglobin (Immediate blood test) | -0.298 | ✓ |  | -0.031 |  |
|  | Glucose (Immediate blood test) | 0.115 | ✓ |  | 0.201 | ✓ |
|  | Cholesterol (mmol/L) | -0.199 | ✓ |  | -0.030 |  |
|  | Apolipoprotein A1 (g/L) | -0.081 |  |  | -0.039 |  |
|  | Apolipoprotein B (g/L) | -0.169 | ✓ |  | -0.038 |  |
|  | Apolipoprotein B/A1 Ratio | -0.075 |  |  | -0.018 |  |
|  | Triglyceride (mmol/L) | -0.008 |  |  | 0.167 | ✓ |

Blood glucose and blood triglyceride were log transformed.

The measure of physiological age was initially implemented among 3178 measurements (804 individuals), of which 2 individual wasn’t included in the present analysis due to a lack of recorded vital status.

Values are Pearson correlation coefficients.

Included in the construction of physiological age**.**

CA, chornological age; BMI, body mass index.

**Supplementary file 1C. List of the 42 items included in the FI and their scoring**

| Item | Scoring |
| --- | --- |
| Hearing status | Perfect=0, Good=0.25, Pretty Good=0.5, Bad=0.75, Deaf or almost deaf=1 |
| Vision status | Perfect=0, Good=0.25, Pretty Good=0.5, Bad=0.75, Blind or almost blind=1 |
| Health prevents from doing things normally would like to do | No=0, Somewhat=0.5, Yes=1 |
| Self-reported general health | Good=0, Mediocre=0.5, Bad=1 |
| Cancer or leukemia | No=0, Yes=1 |
| Rheumatoid arthritis | No=0, Yes=1 |
| Arthritis | No=0, Yes=1 |
| Chronic bronchitis or emphysema | No=0, Yes=1 |
| Cataracts | No=0, Yes=1 |
| Chest pain | No=0, Yes=1 |
| Circulation problems in arms or legs | No=0, Yes=1 |
| Persistent cough | No=0, Yes=1 |
| Diabetes | No=0, Yes=1 |
| Goiter or other gland problems | No=0, Yes=1 |
| Heart failure | No=0, Yes=1 |
| Hypertension | No=0, Yes=1 |
| Kidney disease | No=0, Yes=1 |
| Brittle bones | No=0, Yes=1 |
| Sciatica | No=0, Yes=1 |
| Anemia | No=0, Yes=1 |
| Cerebral hemorrhage or blood clot in brain | No=0, Yes=1 |
| Dizziness | No=0, Yes=1 |
| Gastric ulcer | No=0, Yes=1 |
| Allergies/allergic manifestations | No=0, Yes=1 |
| Asthma | No=0, Yes=1 |
| Shower and bathe^1^ | No problem=0, Needs help=0.5, Cannot=1 |
| Get in and out of bed^1^ | No problem=0, Needs help=0.5, Cannot=1 |
| Dress and undress^1^ | No problem=0, Needs help=0.5, Cannot=1 |
| Self-grooming1 | No problem=0, Needs help=0.5, Cannot=1 |
| Walking1 | No problem=0, Needs help=0.5, Cannot=1 |
| Trouble getting to toilet in time^1^ | No=0, Yes=1 |
| Travel further distances^2^ | Can travel alone=0, Can go by taxi=0.5, Needs helper, special assistance or  doesn’t travel=1 |
| Housework^2^ | No problems=0, Needs help=0.5, Doesn’t do=1 |
| Prepare meals^2^ | Can plan/prepare=0, Can heat up=0.5, Doesn’t cook=1 |
| Manage medications^2^ | No problems=0^,^ Needs help=0.5, Doesn’t do=1 |
| Manage money^2^ | No problems=0, Needs help=0.5, Doesn’t do=1 |
| Use telephone^2^ | Can look up numbers and dial=0, Needs help or doesn’t use phone=1 |
| Grocery shopping^2^ | Can shop=0, Needs help=0.5, Doesn’t shop=1 |
| Feeling lonely^3^ | Never, almost never, rather seldom=0, Quite often, always, almost always=1 |
| Feeling depressed^3^ | Never, almost never or rather seldom=0, Quite often, always, almost always=1 |
| Consider oneself happy and carefree | No=1, Yes=0 |
| Usually feels tired | No=0, Yes=1 |

Note. ^1^from the instrument of Basic Activities of Daily Living, ^2^from the instrument of Instrumental Activities of Daily Living, ^3^ from the Center for Epidemiologic Studies Depression Scale

**Supplementary file 1D. Characteristics of baseline (first available) complete measurements in 288 individuals**

|  | Individuals with complete measurements |
| --- | --- |
| Number of individuals | 288 |
| Women (%) | 56.9 |
| Above primary education (%) | 50.3 |
| BMI (kg/m^2^) | 26.5 (4.1) |
| Smoker (%) | 21.2 |
| Age (year) | 68.2 (9.1) |
| BAs |  |
| Telomere length (T/S ratio) | 0.74 (0.16) |
| DNAmAge (Horvath, year) | 60.5 (10.2) |
| DNAmAge (Hannum, year) | 64.7 (9.3) |
| DNAmPhenoAge (year) | 62.3 (12.1) |
| DNAmGrimAge (year) | 68.7 (8.1) |
| Physiological age (year) | 68.8 (10.6) |
| Cognitive function | 53.5 (10.4) |
| FAI | 47.3 (12.7) |
| FI | 0.09 (0.08) |
| Number of complete measurements (times) | 2.1 (1.1) |

Values are Mean (standard deviation; SD) unless stated otherwise.

Smokers include current and former smokers.

BA, biological age; IPT, In-person testing; DNAmAge, DNA methylation age; FAI, functional aging index; FI, frailty index; BMI, body mass index.

**Supplementary file 1E. Repeated measures correlation coefficients of BAs in 288 individuals**

|  | Age | Telomere length | DNAmAge | | | | Physiological age | Cognitive function | fBio  Age | FI |
| --- | --- | --- | --- | --- | --- | --- | --- | --- | --- | --- |
|  |  |  | Horvath | Hannum | PhenoAge | GrimAge |  |  |  |  |
| Correlations of BAs | | |  |  |  |  |  |  |  |  |
| CA | 1.00 |  |  |  |  |  |  |  |  |  |
| Telomere length | -0.11 | 1.00 |  |  |  |  |  |  |  |  |
| DNAmAge (Horvath) | 0.53 | -0.09 | 1.00 |  |  |  |  |  |  |  |
| DNAmAge (Hannum) | 0.64 | -0.16 | 0.56 | 1.00 |  |  |  |  |  |  |
| DNAmPhenoAge | 0.53 | -0.07 | 0.27 | 0.41 | 1.00 |  |  |  |  |  |
| DNAmGrimAge | 0.85 | -0.09 | 0.44 | 0.59 | 0.49 | 1.00 |  |  |  |  |
| Physiological age | 0.87 | -0.07 | 0.48 | 0.58 | 0.47 | 0.74 | 1.00 |  |  |  |
| Cognitive function | -0.45 | -0.10 | -0.25 | -0.24 | -0.30 | -0.42 | -0.42 | 1.00 |  |  |
| FAI | 0.54 | -0.06 | 0.32 | 0.31 | 0.28 | 0.43 | 0.49 | -0.50 | 1.00 |  |
| FI | 0.45 | -0.06 | 0.25 | 0.26 | 0.24 | 0.36 | 0.39 | -0.29 | 0.48 | 1.00 |
| Correlations of BA residuals | |  |  |  |  |  |  |  |  |  |
| CA | 1.00 |  |  |  |  |  |  |  |  |  |
| Telomere length | 0.00 | 1.00 |  |  |  |  |  |  |  |  |
| DNAmAge (Horvath) | -0.02 | -0.04 | 1.00 |  |  |  |  |  |  |  |
| DNAmAge (Hannum) | -0.04 | -0.12 | 0.35 | 1.00 |  |  |  |  |  |  |
| DNAmPhenoAge | 0.00 | -0.01 | -0.02 | 0.11 | 1.00 |  |  |  |  |  |
| DNAmGrimAge | 0.07 | -0.01 | 0.00 | 0.13 | 0.09 | 1.00 |  |  |  |  |
| Physiological age | -0.10 | 0.04 | 0.06 | 0.05 | 0.02 | 0.03 | 1.00 |  |  |  |
| Cognitive function | 0.03 | -0.17 | -0.04 | 0.02 | -0.06 | -0.07 | -0.12 | 1.00 |  |  |
| FAI | 0.01 | 0.00 | 0.07 | -0.04 | -0.02 | -0.08 | 0.07 | -0.32 | 1.00 |  |
| FI | -0.07 | -0.03 | 0.05 | -0.01 | -0.02 | -0.04 | 0.02 | -0.10 | 0.31 | 1.00 |

BA, biological age; DNAmAge, DNA methylation age; FAI, functional aging index; FI, frailty index; CA, chronological age.

**Supplementary file 1F. Survival analyses of baseline (first available) BA residuals with the risk of all-cause mortality in 845 individuals (one-BA models)**

| BA residuals | Number of individuals | Number of deaths | Median follow-up time (year) | Model 1 | Model 2 |
| --- | --- | --- | --- | --- | --- |
| Telomere length | 636 | 389 | 15.8 | 0.98 (0.90, 1.08) | 1.01 (0.92, 1.10) |
| DNAmAge (Horvath) | 387 | 240 | 16.1 | 1.12 (0.99, 1.27) | 1.16 (1.02, 1.32) |
| DNAmAge (Hannum) | 387 | 240 | 16.1 | 1.18 (1.03, 1.36) | 1.15 (0.99, 1.32) |
| DNAmPhenoAge | 387 | 240 | 16.1 | 1.19 (1.07, 1.33) | 1.23 (1.09, 1.38) |
| DNAmGrimAge | 387 | 240 | 16.1 | 1.32 (1.09, 1.59) | 1.34 (1.12, 1.60) |
| Physiological age | 802 | 543 | 18.7 | 1.10 (1.02, 1.20) | 1.10 (1.00, 1.21) |
| Cognitive function | 829 | 570 | 19.2 | 0.82 (0.75, 0.90) | 0.86 (0.77, 0.94) |
| FAI | 739 | 481 | 17.9 | 1.27 (1.14, 1.42) | 1.24 (1.10, 1.38) |
| FI | 756 | 498 | 17.7 | 1.33 (1.21, 1.47) | 1.32 (1.20, 1.46) |

Values are Hazard Ratios (95% Confidence Interval) [HR (95%CI)] unless stated otherwise.

HRs (95%CIs) in each column refer to the relative risks associated with one-SD increase in the level of BA of nine different models with one corresponding BA residual being the predictor of the mortality risk. Model 1 is the uni-variate survival model with only one BA residual taken into account. Model 2 is the multi-variate survival model, in which common risk factors (sex, education attainment, smoking status, and BMI) were additionally adjusted for on the basis of Model 1. All models were stratified by participants’ birth year (in 10-year interval). Attained age was used as the time-scale and thus age was inherently adjusted for.

BA, biological age; DNAmAge, DNA methylation age; FAI, functional aging index; FI, frailty index.

**Supplementary file 1G. Survival analyses of baseline BAs with the risk of all-cause mortality in 845 individuals stratified by sex (one-BA models).**

| BAs | Number of individuals | Number of deaths | Median follow-up time (year) | Model 1 | Model 2 |
| --- | --- | --- | --- | --- | --- |
| Men |  |  |  |  |  |
| Telomere length | 264 | 170 | 14.6 | 0.98 (0.86, 1.10) | 1.01 (0.90, 1.14) |
| DNAmAge (Horvath) | 155 | 95 | 15.2 | 1.28 (1.01, 1.61) | 1.45 (1.14, 1.83) |
| DNAmAge (Hannum) | 155 | 95 | 15.2 | 1.04 (0.84, 1.29) | 1.05 (0.82, 1.34) |
| DNAmPhenoAge | 155 | 95 | 15.2 | 1.00 (0.78, 1.27) | 1.04 (0.81, 1.33) |
| DNAmGrimAge | 155 | 95 | 15.2 | 1.19 (0.86, 1.64) | 1.21 (0.88, 1.66) |
| Physiological age | 330 | 235 | 17.7 | 1.12 (0.83, 1.50) | 1.20 (0.88, 1.63) |
| Cognitive function | 335 | 242 | 18.6 | 0.94 (0.83, 1.08) | 0.97 (0.84, 1.12) |
| FAI | 303 | 210 | 16.8 | 1.27 (1.04, 1.56) | 1.22 (0.99, 1.50) |
| FI | 307 | 214 | 16.9 | 1.16 (0.97, 1.38) | 1.13 (0.95, 1.35) |
| Women |  |  |  |  |  |
| Telomere length | 372 | 219 | 16.5 | 1.01 (0.89, 1.14) | 1.03 (0.90, 1.18) |
| DNAmAge (Horvath) | 232 | 145 | 16.3 | 1.03 (0.86, 1.24) | 1.08 (0.90, 1.30) |
| DNAmAge (Hannum) | 232 | 145 | 16.3 | 1.34 (1.06, 1.70) | 1.38 (1.08, 1.76) |
| DNAmPhenoAge | 232 | 145 | 16.3 | 1.35 (1.15, 1.59) | 1.45 (1.22, 1.72) |
| DNAmGrimAge | 232 | 145 | 16.3 | 1.66 (1.26, 2.19) | 1.65 (1.25, 2.16) |
| Physiological age | 472 | 308 | 19.2 | 1.16 (0.98, 1.37) | 1.18 (0.98, 1.41) |
| Cognitive function | 494 | 328 | 19.9 | 0.73 (0.64, 0.84) | 0.75 (0.65, 0.86) |
| FAI | 436 | 271 | 18.7 | 1.38 (1.15, 1.64) | 1.35 (1.14, 1.61) |
| FI | 449 | 284 | 18.5 | 1.43 (1.24, 1.65) | 1.43 (1.25, 1.64) |

Values are Hazard Ratios (95% Confidence Interval) [HR (95%CI)] unless stated otherwise.

HRs (95%CIs) refer to the relative risks associated with one-SD increase in the level of BA in each subgroups. Model 1 is the uni-variate survival model with only one BA taken into account. Model 2 is the multi-variate survival model, in which common risk factors (sex, education attainment, smoking status, and BMI) were additionally adjusted for on the basis of Model 1. All models were stratified by participants’ birth year (in 10-year interval). Attained age was used as the time-scale and thus age was inherently adjusted for.

BA, biological age; DNAmAge, DNA methylation age; FAI, functional aging index; FI, frailty index.

**Supplementary file 1H. Survival analyses of baseline BAs with the risk of all-cause mortality in 845 individuals stratified by age group (one-BA models).**

| BAs | Number of individuals | Number of deaths | Median follow-up time (year) | Model 1 | Model 2 |
| --- | --- | --- | --- | --- | --- |
| Baseline age ≤65 years |  |  |  |  |  |
| Telomere length | 222 | 63 | 18.7 | 0.88 (0.70, 1.11) | 0.96 (0.77, 1.20) |
| DNAmAge (Horvath) | 128 | 30 | 20 | 1.33 (0.76, 2.30) | 1.10 (0.59, 2.03) |
| DNAmAge (Hannum) | 128 | 30 | 20 | 1.58 (0.88, 2.84) | 1.41 (0.81, 2.45) |
| DNAmPhenoAge | 128 | 30 | 20 | 1.41 (1.04, 1.91) | 1.41 (0.94, 2.12) |
| DNAmGrimAge | 128 | 30 | 20 | 2.52 (1.57, 4.06) | 1.96 (1.03, 3.73) |
| Physiological age | 415 | 183 | 25 | 1.16 (0.90, 1.50) | 1.06 (0.77, 1.45) |
| Cognitive function | 465 | 223 | 25.3 | 0.89 (0.77, 1.03) | 0.88 (0.76, 1.03) |
| FAI | 353 | 134 | 24.3 | 1.57 (1.16, 2.11) | 1.58 (1.15, 2.15) |
| FI | 356 | 138 | 24.6 | 1.52 (1.20, 1.92) | 1.41 (1.10, 1.83) |
| Baseline age > 65 years | |  |  |  |  |
| Telomere length | 414 | 326 | 12.3 | 0.97 (0.87, 1.07) | 1.01 (0.92, 1.12) |
| DNAmAge (Horvath) | 259 | 210 | 13.3 | 1.13 (0.97, 1.30) | 1.16 (0.99, 1.35) |
| DNAmAge (Hannum) | 259 | 210 | 13.3 | 1.22 (1.03, 1.44) | 1.17 (0.97, 1.41) |
| DNAmPhenoAge | 259 | 210 | 13.3 | 1.18 (1.03, 1.36) | 1.21 (1.03, 1.42) |
| DNAmGrimAge | 259 | 210 | 13.3 | 1.43 (1.11, 1.85) | 1.35 (1.04, 1.77) |
| Physiological age | 387 | 360 | 14.9 | 1.10 (0.92, 1.30) | 1.14 (0.95, 1.35) |
| Cognitive function | 364 | 347 | 15.3 | 0.79 (0.70, 0.90) | 0.79 (0.69, 0.92) |
| FAI | 386 | 347 | 14.8 | 1.13 (0.98, 1.30) | 1.21 (1.03, 1.42) |
| FI | 400 | 360 | 14.8 | 1.23 (1.09, 1.39) | 1.30 (1.15, 1.46) |

Values are Hazard Ratios (95% Confidence Interval) [HR (95%CI)] unless stated otherwise.

HRs (95%CIs) refer to the relative risks associated with one-SD increase in the level of BA in each subgroups. Model 1 is the uni-variate survival model with only one BA taken into account. Model 2 is the multi-variate survival model, in which common risk factors (sex, education attainment, smoking status, and BMI) were additionally adjusted for on the basis of Model 1. All models were stratified by participants’ birth year (in 10-year interval). Attained age was used as the time-scale and thus age was inherently adjusted for.

BA, biological age; DNAmAge, DNA methylation age; FAI, functional aging index; FI, frailty index.

**Supplementary file 1I. Survival analyses of baseline BAs with the risk of all-cause mortality in 845 individuals stratified by smoking status (one-BA models).**

| BAs | Number of individuals | Number of deaths | Median follow-up time (year) | Model 1 | Model 2 |
| --- | --- | --- | --- | --- | --- |
| Non-smokers at baseline | |  |  |  |  |
| Telomere length | 495 | 304 | 15.7 | 0.98 (0.89, 1.09) | 1.01 (0.91, 1.12) |
| DNAmAge (Horvath) | 303 | 188 | 16.2 | 1.10 (0.94, 1.30) | 1.05 (0.89, 1.25) |
| DNAmAge (Hannum) | 303 | 188 | 16.2 | 1.29 (1.07, 1.55) | 1.18 (0.98, 1.43) |
| DNAmPhenoAge | 303 | 188 | 16.2 | 1.25 (1.08, 1.44) | 1.29 (1.10, 1.50) |
| DNAmGrimAge | 303 | 188 | 16.2 | 1.41 (1.08, 1.84) | 1.31 (1.01, 1.70) |
| Physiological age | 609 | 415 | 18.4 | 1.16 (1.00, 1.35) | 1.12 (0.95, 1.31) |
| Cognitive function | 618 | 429 | 19.2 | 0.83 (0.74, 0.93) | 0.84 (0.74, 0.96) |
| FAI | 561 | 369 | 17.7 | 1.20 (1.03, 1.41) | 1.27 (1.09, 1.50) |
| FI | 577 | 385 | 17.5 | 1.29 (1.14, 1.46) | 1.32 (1.17, 1.50) |
| Current or ex-smokers at baseline | |  |  |  |  |
| Telomere length | 141 | 85 | 16.3 | 0.98 (0.78, 1.24) | 1.09 (0.84, 1.40) |
| DNAmAge (Horvath) | 84 | 52 | 15.5 | 1.31 (0.98, 1.75) | 1.34 (0.94, 1.91) |
| DNAmAge (Hannum) | 84 | 52 | 15.5 | 0.98 (0.68, 1.43) | 1.09 (0.77, 1.54) |
| DNAmPhenoAge | 84 | 52 | 15.5 | 1.08 (0.69, 1.68) | 1.32 (0.83, 2.08) |
| DNAmGrimAge | 84 | 52 | 15.5 | 1.36 (0.88, 2.10) | 1.63 (1.12, 2.38) |
| Physiological age | 193 | 128 | 19.5 | 1.15 (0.81, 1.63) | 1.17 (0.79, 1.73) |
| Cognitive function | 211 | 141 | 19.2 | 0.84 (0.70, 1.02) | 0.87 (0.69, 1.09) |
| FAI | 178 | 112 | 18.0 | 1.21 (0.94, 1.56) | 1.30 (0.98, 1.72) |
| FI | 179 | 113 | 18.1 | 1.26 (1.03, 1.53) | 1.34 (1.10, 1.62) |

Values are Hazard Ratios (95% Confidence Interval) [HR (95%CI)] unless stated otherwise.

HRs (95%CIs) refer to the relative risks associated with one-SD increase in the level of BA in each subgroups. Model 1 is the uni-variate survival model with only one BA taken into account. Model 2 is the multi-variate survival model, in which common risk factors (sex, education attainment, smoking status, and BMI) were additionally adjusted for on the basis of Model 1. All models were stratified by participants’ birth year (in 10-year interval). Attained age was used as the time-scale and thus age was inherently adjusted for.

BA, biological age; DNAmAge, DNA methylation age; FAI, functional aging index; FI, frailty index.

**Supplementary file 1J. Survival analyses of baseline BAs with the risk of all-cause mortality in 288 individuals with complete measurements (one-BA models)**

|  |  | BAs | |  | BA residuals | |
| --- | --- | --- | --- | --- | --- | --- |
|  |  | Model 1 | Model 2 |  | Model 1 | Model 2 |
| Telomere length |  | 0.91 (0.79, 1.05) | 0.96 (0.83, 1.11) |  | 0.93 (0.81, 1.07) | 0.95 (0.82, 1.10) |
| DNAmAge (Horvath) |  | 1.08 (0.90, 1.29) | 1.17 (0.96, 1.43) |  | 1.10 (0.94, 1.30) | 1.21 (1.01, 1.45) |
| DNAmAge (Hannum) |  | 1.24 (0.99, 1.54) | 1.19 (0.93, 1.52) |  | 1.27 (1.06, 1.53) | 1.24 (1.02, 1.50) |
| DNAmPhenoAge |  | 1.20 (0.98, 1.46) | 1.25 (1.01, 1.54) |  | 1.22 (1.04, 1.44) | 1.26 (1.07, 1.48) |
| DNAmGrimAge |  | 1.44 (1.08, 1.93) | 1.24 (0.93, 1.67) |  | 1.34 (1.05, 1.70) | 1.35 (1.07, 1.71) |
| Physiological age |  | 0.90 (0.71, 1.14) | 0.99 (0.75, 1.30) |  | 1.01 (0.88, 1.15) | 1.06 (0.90, 1.24) |
| Cognitive function |  | 0.86 (0.73, 1.01) | 0.89 (0.76, 1.04) |  | 0.82 (0.70, 0.97) | 0.86 (0.74, 1.01) |
| FAI |  | 1.22 (1.01, 1.46) | 1.25 (1.04, 1.51) |  | 1.34 (1.14, 1.57) | 1.30 (1.10, 1.52) |
| FI |  | 1.37 (1.16, 1.63) | 1.46 (1.20, 1.77) |  | 1.50 (1.28, 1.75) | 1.53 (1.28, 1.82) |

Values are Hazard Ratios (95% Confidence Interval) [HR (95%CI)] unless stated otherwise.

Supplementary Table 7 replicated the analysis in Table 4 and Supplementary Table 6 in individuals with complete measurements.

HRs (95%CIs) in each column refer to the relative risks associated with one-SD increase in the level of BA of nine different models with one corresponding BA or BA residual being the predictor of the mortality risk. Model 1 is the uni-variate survival model with only one BA taken into account. Model 2 is the multi-variate survival model, in which common risk factors (sex, education attainment, smoking status, and BMI) were additionally adjusted for on the basis of Model 1. All models were stratified by participants’ birth year (in 10-year interval). Attained age was used as the time-scale and thus age was inherently adjusted for.

BA, biological age; DNAmAge, DNA methylation age; FAI, functional aging index; FI, frailty index.

**Supplementary file 1K. Survival analysis with additional adjustment for previous diseases**

| BAs | One-BA models | Nine-BA model |
| --- | --- | --- |
| Telomere length | 1.03 (0.92, 1.14) | 1.05 (0.89, 1.24) |
| DNAmAge (Horvath) | 1.10 (0.94, 1.29) | 1.28 (1.05, 1.56) |
| DNAmAge (Hannum) | 1.11 (0.92, 1.34) | 0.96 (0.31, 3.02) |
| DNAmPhenoAge | 1.19 (1.01, 1.40) | 1.12 (0.86, 1.44) |
| DNAmGrimAge | 1.25 (0.96, 1.62) | 1.42 (1.09, 1.86) |
| Physiological age | 1.01 (0.85, 1.19) | 0.97 (0.85, 1.12) |
| Cognitive function | 0.85 (0.75, 0.95) | 1.01 (0.84, 1.20) |
| FAI | 1.19 (1.03, 1.38) | 1.11 (0.85, 1.45) |
| FI | 1.24 (1.10, 1.40) | 1.50 (1.26, 1.79) |

Sensitivity analyses additionally adjusted for the presence of heart failure, stroke, diabetes, and cancer at baseline (first available measurement) based on the models specified in the Model 2 of Table 4 and the Model 2 of Table 5.
